# Endoscopy within 7 days after detecting high calprotectin levels can be useful for therapeutic decision-making in ulcerative colitis

**DOI:** 10.1097/MD.0000000000027065

**Published:** 2021-08-27

**Authors:** Ho Min Yong, Sung-Jo Park, Seong Ran Jeon, Heesu Park, Hyun Gun Kim, Tae Hee Lee, Junseok Park, Jin-Oh Kim, Joon Seong Lee, Bong Min Ko, Hyeon Jeong Goong, Suyeon Park

**Affiliations:** a Digestive Disease Center, Institute for Digestive Research, Seoul, Korea; b Department of biostatistics, Soonchunhyang University College of Medicine, Seoul, Korea.

**Keywords:** Endoscopy, Fecal calprotectin, Therapeutic plan, Time interval, Ulcerative colitis

## Abstract

The aim of this study was to assess the appropriate time interval to identify the association between the fecal calprotectin (FC) test and endoscopic activity, and to evaluate whether the time interval affects the therapeutic plan adjustment in patients with ulcerative colitis (UC).

This study included 103 patients who underwent FC tests and endoscopic examinations within the past three months. The FC test results classified cases into three groups as follows: moderate to severe (>200, >250, or >300 μg/g), mild (100–200, 100–250, or 100–300 μg/g), and inactive (<100 μg/g) activity. The Mayo endoscopic subscore was used to determine endoscopic activity. Therapeutic plan adjustment included the addition or increased dosage of anti-inflammatory drugs, steroids, immunomodulators, and biologics.

Using the cutoff value for FC of 200 μg/g, the appropriate time interval for dividing the association and non-association between Mayo endoscopic subscore and FC was 7 days (sensitivity, 74.4%; specificity, 50.0%; area under the curve [AUC], 0.6032). When using FC 250 or 300 μg/g, the appropriate time interval was 5.5 days, with a sensitivity of 71.7% and specificity of 49.1 (AUC 0.5862) in FC 250 μg/g, a sensitivity of 69.6%, and a specificity of 47.4 (AUC 0.5549) for FC 300 μg/g. Therapeutic plans changed in 29.1% of patients. In patients with shorter intervals (≤7 days) between the FC test and endoscopy, significant therapeutic plan adjustments were observed in patients with UC (36.5% vs. 17.5%, *P* = .047).

Although the need for endoscopy within 7 days after detecting high FC (≥ 200 μg/g) was not statistically supported, endoscopy within a shorter interval (≤7 days) in UC patients with high FC can help determine the therapeutic plan.

## 1 Introduction

Ulcerative colitis (UC) is a chronic intestinal disease characterized by recurrence and repetition of exacerbations of bowel inflammation.^[1,2]^ In Asia, its incidence and prevalence are increasing.^[3,4]^ Traditionally, the therapeutic goal for UC has been to maintain clinical remission by controlling symptoms. Recently, the therapeutic target has been shifted to a more objective parameter, such as mucosal healing, to be associated with continuous remission and reduction of hospitalization or operation for UC.^[5–7]^ Although the role of endoscopy is essential for mucosal inflammation assessment, it is uncomfortable, invasive, and expensive, and may induce major complications such as colon perforation.

On the other hand, fecal calprotectin (FC) analysis is deemed to be more easily accessible and noninvasive than endoscopy.^[8]^ FC, which reflects neutrophil migration into the gut lumen, has been used as a surrogate marker to predict endoscopic activity in UC patients.^[9–11]^ In addition, a recent meta-analysis showed that FC has high sensitivity (75%), specificity (77%), and positive likelihood ratio (3.45) in predicting UC relapse in adult.^[12]^

Nevertheless, endoscopy is still considered the gold standard for the evaluation of intestinal mucosal inflammation because changes in therapeutic options are not recommended based only on FC measurement in symptomatic UC patients.^[13,14]^ The timing of FC testing should be at the same time as the endoscopy to more accurately determine the association between the FC test and endoscopic disease activity, and vice versa. In practice, however, both tests cannot be easily performed simultaneously. Although the role of FC in predicting recurrence in UC patients and the relationship between high levels of FC and high levels of intestinal inflammation have been almost established, to the best of our knowledge no study has examined the appropriate time interval to confirm the association between the FC measurement and endoscopic disease activity in patients with UC. Therefore, the aim of this study was to assess the appropriate time interval to confirm the association between the FC test and endoscopic activity, and to evaluate whether the time interval affects the therapeutic plan adjustment in patients with UC.

## 2 Methods

### 2.1 Subjects

This study was retrospectively conducted on 103 cases (79 patients) with FC tests and endoscopic examinations performed within three months of each other, between February 2015 and September 2019 at the Soonchunhyang University Hospital. Patients with previous surgical bowel operation, indeterminate or nonspecific colitis, liver disease, combined gastrointestinal tract infection, malignancy, hematologic disorder, pulmonary or heart disease, autoimmune disease, or kidney disease were excluded. This study was approved by our institutional review board (SCHUH 2019–12–003). Patient consent to participate was waived in accordance with the institutional review board. The study protocol followed the ethical guidelines of the 1975 Declaration of Helsinki and was approved by the institution's human research committee.

### 2.2 Laboratory values

EliA^TM^ Calprotectin 2 (Phadia AB, Uppsala, Sweden) kits were used to measure FC levels. Fecal samples were collected at home or in a hospital. When collected at home, patients were instructed to refrigerate their fecal samples (2–8°C). The fecal samples were stored in aliquots at −20°C or below until analysis using the fluorescence immunoassay method per manufacturer's instructions was performed. The EliA Calprotectin 2 kit measures FC levels in the range of 3.8 – ≥ 6,000 μg/g. As stated by the manufacturer, the cutoff level of FC, representing a positive value, was ≥ 50 μg/g. Based on previous studies.^[13,15]^ the FC test results classified cases as follows: moderate to severe (>200 μg/g, >250 μg/g, or >300 μg/g), mild (100–200 μg/g, 100–250 μg/g, or 100–300 μg/g), and inactive (<100 μg/g) activity. FC cutoff values of 200 μg/g, 250 μg/g and 300 μg/g were denoted as FC1, FC2, and FC3, respectively.

### 2.3 Assessment of activities and therapeutic plan adjustment

The UC clinical phenotype was classified according to the Montreal classification.^[16]^ The Mayo score was used to evaluate the activity of UC disease. The Mayo endoscopic subscore (MES) was used to assess endoscopic disease activity. MES has been classified into four categories; 0, inactive; 1, mild (erythema, mild friability and decreased vascular pattern); 2, moderate (marked erythema, friability, erosions, and lack of vascular pattern,); and 3, severe (ulcerations and spontaneous bleeding).^[17]^ In left-sided or pancolitis, the score was given based on the area with the most severe inflammatory segment. Two board-certified endoscopy experts (S. R. J and H. G. K), who were blinded to the clinical and laboratory information, reviewed the endoscopic images. If disagreement occurred between the two reviewers, the endoscopic activity score was determined based on the consensus of the two reviewers. Findings of FC tested in one month and endoscopy performed in three months were used. The median time interval between endoscopy and FC measurement was 1 (interquartile range [IQR] 0–14) days.

Therapeutic plan adjustment was defined as the addition or increased dosage of anti-inflammatory drugs, steroids, immunomodulators, and biologics after endoscopy following the FC test in patients with UC.

### 2.4 Statistical analysis

Categorical and continuous variables were compared using the chi-squared test and two-tailed Student's t-test, respectively. Correlations among FC, disease activity, and endoscopic activity were assessed using Spearman's correlation coefficient. Receiver operating characteristic (ROC) curve analysis was used to determine the appropriate time interval to assess the association between the FC test and endoscopic disease activity in patients with UC. The sensitivity, specificity, accuracy, positive predictive value (PPV), and negative predictive value (NPV) according to the time interval between FC measurement and endoscopy were assessed using ROC curve analyses. After assessing the connection between the FC test and MES, we used the Youden index method to estimate the appropriate time interval between examinations. Finally, the area under the curve (AUC) was compared for each criterion (>200 μg/g, >250 μg/g, or >300 μg/g) using Delong's test. Statistical significance was set at p < 0.05. The Bonferroni correction method was used to solve the multiple testing problem. All statistical analyzes were performed using version 3.6.1 (“pROC” and “Optimal Cut points” packages) and SPSS 19.0 (SPSS Inc., Chicago, IL).

## 3 Results

### 3.1 Subject characteristics

Eighty-six patients with UC who underwent endoscopy were initially enrolled in the study. Seven patients were excluded (five for unexamined fecal samples, one for having a concomitant gastrointestinal infection, and one for bowel resection). Finally, 79 patients with UC were enrolled in the study. The median age of patients with UC was 39 years (IQR 25–51 years), and 64.5% (51/79) were men. The median Mayo scores at the time of diagnosis and FC measurements were 6 (IQR 4–6) and 4 (IQR 2–7), respectively. The median FC level was 285.8 (77.0–749.1). The clinical characteristics of patients with UC, including laboratory tests and medications, at FC measurements are summarized in Table 1.

**Table 1:** Baseline characteristics at diagnosis in patients with ulcerative colitis.

Variable	UC (n = 79)
Male, n (%)	51 (64.5)
Previous op. history, n (%)	6 (7.6)
Appendectomy / perianal op / others^∗^	0 / 5 (83.3) / 1 (16.7)
Age at diagnosis (years), median (IQR)	42 (24–51)
A1 (<17 yrs), n (%)	3 (3.8)
A2 (17–40 yr), n (%)	39 (49.4)
A3 (> 40 yr), n (%)	37 (46.8)
Disease extension at diagnosis, n (%)	
E1 (proctitis)	33 (41.8)
E2 (left-sided colitis)	21 (26.6)
E3 (extensive colitis)	25 (31.6)
Disease activity at diagnosis	
Clinical remission (Mayo score 0–2)	5 (6.3)
Mild activity (Mayo score 3–5)	32 (40.5)
Moderate activity (Mayo score 6–10)	35 (44.3)
Severe activity (Mayo score 11–12)	7 (8.9)
Mayo score at diagnosis, median (IQR)	6 (4–9)
Disease activity at FC measurement^∗∗^	
Clinical remission (Mayo score 0–2)	30 (29.1)
Mild activity (Mayo score 3–5)	34 (33.0)
Moderate activity (Mayo score 6–10)	33 (32.0)
Severe activity (Mayo score 11–12)	6 (5.8)
Mayo score at FC measurement, median (IQR) FC level (μg/g)^∗∗^, median (IQR)	5 (2–7) 285.8 (77.0–749.1)
Laboratory tests at FC measurement^∗∗^, median (IQR)	
White blood cell count (/uL)	6500 (5500–7800)
Hemoglobin (g/dL)	13.8 (12.7–14.8)
Hematocrit (%)	41.9 (38.1–44.8)
Erythrocyte sedimentation rate (mm/h)	31 (15.0–46.5)
C-reactive protein (mg/dL)	0.11 (0.04–0.37)
Albumin (g/dL)	4.6 (4.3–4.8)
Medication use at FC measurement^∗∗^ n (%)	
5-ASA	41 (39.8)
5-ASA + topical 5-ASA	39 (37.9)
5-ASA + topical 5-ASA + steroid	4 (3.9)
5-ASA + steroid + AZA	3 (2.9)
5-ASA + topical 5-ASA + AZA	3 (2.9)
5-ASA + topical 5-ASA + AZA + steroid	3 (2.9)
5-ASA + topical 5-ASA + AZA + biologics	3 (2.9)
Biologics	2 (1.9)
5-ASA + steroid	2 (1.9)
5-ASA + topical steroid	2 (1.9)
5-ASA + AZA	1 (1.0)

Variables are presented as mean ± SD or n (%).

∗ Others: hysterectomy, transurethral resection of bladder.

∗∗ Variables are calculated in 103 cases. 5-ASA = 5-amicosalicylic acid, AZA = azathiorpine, FC = fecal calprotectin, IQR = interquartile range, Op = operation, UC = ulcerative colitis.

### 3.2 ROC curve and corresponding AUC analysis

In the FC cutoff value of 200 μg/g (FC1), the appropriate time interval for determining the association between FC and MES was 7 days (sensitivity 74.4%, specificity 50.0%, accuracy 61.2%, PPV 55.6%, NPV 70.0%, and AUC 0.6032. 95% confidence interval [CI], 0.4779 – 0.6896). In the FC cutoff values of 250 (FC2) or 300 μg/g (FC3), which distinguishes between mild and moderate, the appropriate time interval was 5.5 days for both levels (FC 250, sensitivity 71.7%, specificity 49.1%, accuracy 59.2%, PPV 53.2%, NPV 68.3%, AUC 0.5862, 95% CI 0.4728 – 0.6884; FC 300, sensitivity 69.6%, specificity 47.4%, accuracy 57.3%, PPV 51.6%, and NPV 65.9%, AUC 0.5549, 95% CI 0.4668 – 0.6513) (Table 2). Pairwise comparison of AUC were made in three cases: *P* = .826 for FC1 vs. FC2; *P* = .536 for FC1 vs. FC3; *P* = .688 for FC2 vs. FC3. Therefore, the FC1 method with the highest AUC was finally selected in this study because *P* = 1.000 in all cases after Bonferroni calibration.

**Table 2:** The receiver operator characteristic curve and area under the curve according to the time interval between the fecal calprotectin (FC) measurement and endoscopy for determining the association between FC and Mayo endoscopic subscore.

Cut-off value of FC (μg/g)	Interval (days)	AUC (95% CI)	Sensitivity	Specificity	Accuracy	PPV	NPV
200	7.0	0.6032 (0.4779–0.6896)	0.7447	0.5000	0.6117	0.5556	0.7000
250	5.5	0.5862 (0.4728–0.6884)	0.7174	0.4912	0.5922	0.5323	0.6829
300	5.5	0.5549 (0.4668–0.6513)	0.6957	0.4737	0.5728	0.5161	0.6585

AUC = area under the curve, CI = confidence interval, FC = fecal calprotectin, NPV = negative predictive value, PPV = positive predictive value.

### 3.3 FC level and therapeutic plan adjustment according to the time interval between the FC test and endoscopy

**Figure 1:**
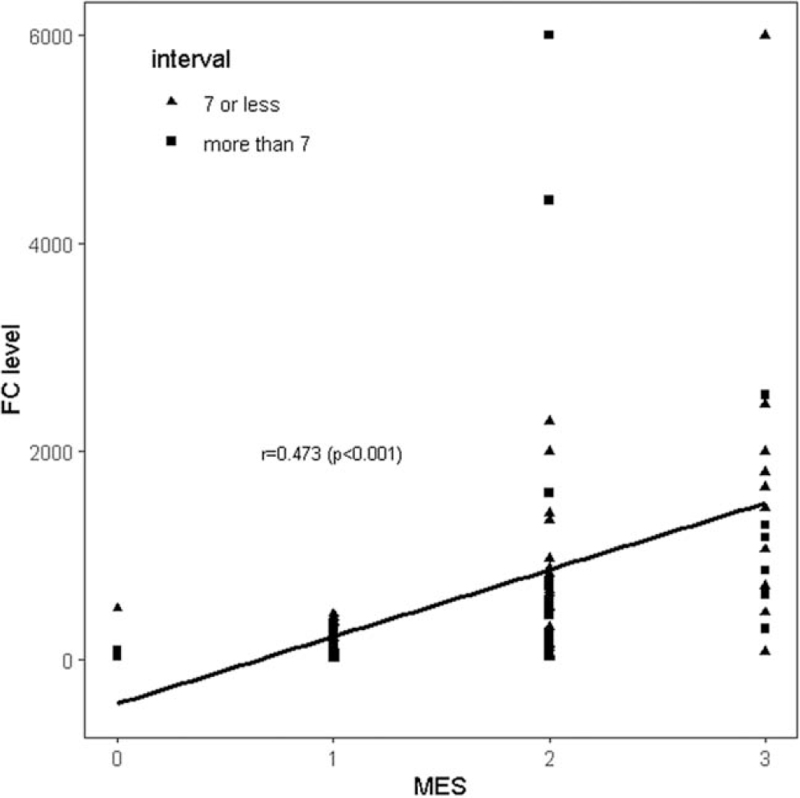
Correlations of fecal calprotectin (FC) according to ulcerative colitis (UC) activity (Mayo endoscopic subscore, MES) and the time interval between the FC measurement and endoscopy in patients with UC. Regardless of the time interval between the FC test and endoscopy, an increase in FC level was correlated with endoscopic severity by MES.

FC level (150.79 ± 132.95 μg/g vs. 1063.56 ± 1257.74 μg/g, *P* < .001) was significantly increased in moderate to severe UC cases than in the inactive to mild UC cases. Although MES and FC (r = 0.473, *P* < .001) were weakly correlated in overall enrolled patients, an increase in FC level was correlated with endoscopic severity by MES, regardless of the time interval between the FC test and endoscopy (Fig. 1).

Therapeutic plans were changed in 29.1% of the total enrolled patients. In patients with shorter intervals (≤7 days) between the FC test and endoscopy, more significant therapeutic plan adjustments were observed with longer intervals (>7 days) in patients with UC (36.5% vs. 17.5%, *P* = .047) (Table 3).

**Table 3:** Therapeutic plan adjustment for patients according to the time interval after performing endoscopy in ulcerative colitis with high calprotectin.

	Shorter intervals (≤7 days) (n = 63)	Longer intervals (>7 days) (n = 40)	*P* value
No change	40 (63.5)	33 (82.5)	.047
Therapeutic plan adjustment	23 (36.5)	7 (17.5)	
Add and/or increased dosage of 5-ASA	17 (27.0)	5 (12.5)	
Add of steroids	2 (3.2)	2 (5.0)	
Add and/or increased dosage of IM	1 (1.6)	0 (0.0)	
Add and/or increased dosage of 5-ASA and steroids	1 (1.6)	0 (0.0)	
Add and/or increased dosage of 5-ASA and IM	2 (3.2)	0 (0.0)	

Variables are presented as n (%). 5-ASA = 5-amicosalicylic acid, IM = immunomodulator.

## 4 Discussion

This study aimed to analyze the appropriate time interval to confirm the association between the FC test and endoscopic activity in patients with UC, and to identify when to perform endoscopy for adjustment of the therapeutic plan in UC patients with high FC. To our knowledge, although all previous studies focused on FC testing only as a role for the diagnosis and clinical follow-up of inflammatory bowel disease (IBD), no study has examined the appropriate time interval to confirm the association between the FC test and endoscopic disease activity in patients with UC. In addition, this study investigated whether the therapeutic plan was changed according to the time interval between the FC test and endoscopic examination in UC patients with high FC. Although we found that the appropriate time interval for determining the association between FC and MES was 7 days in UC patients with FC 200 μg/g or more, this result did not have statistically significant implications. However, we found that endoscopy within a shorter interval (≤7 days) in UC patients with high FC (≥ 200 μg/g) can help determine the therapeutic plan.

Because an increase in the FC level during serial measurements is a marker of increased mucosal activity and may predict the clinical relapse of IBD, FC measurements are performed before initiating IBD therapy and during regular follow-up every three or six months throughout during treatment.^[13,18]^ When the FC level is increased, endoscopy is considered to decide whether to escalate or to keep the current treatment. According to a previous study, FC could be a useful tool to identify endoscopically active UC and be helpful to monitor disease activity and decide on treatment escalation.^[19]^ Ideally, accurate mucosal inflammation can be evaluated by performing endoscopy on the same day as the increase in the FC level was observed. A recent guideline recommended that fecal samples not be stored for three days or more at room temperature prior to analysis.^[18]^ In real world practice, however, FC measurement and endoscopy are often not performed on the same day because of a lack of fecal sampling, collection delays, patients’ personal circumstances, or institutional practice patterns. In our study, endoscopy was performed within three days after FC measurement in 59% of cases (61/103) and at the same time after FC measurement in only 37% (38/103).

In a study, the authors illustrated that a 57 μg/g cutoff value for FC predicted endoscopic mucosal inflammation as defined by the modified Baron Index ≥ 2 with a specificity and sensitivity of 90% and 91%, respectively.^[20]^ However, another study revealed that FC with a 250 μg/g cutoff level had a specificity and sensitivity of 100% and 71%, respectively, for active mucosal inflammation as defined by a MES ≥ 1.^[21]^ Likewise, optimal cutoff values for predicting mucosal inflammation has been reported from 50–250 μg/g in UC patients due to the use of various endoscopic activity indices, type and location of the disease, and variability between different assay methods or in different fecal samples from similar patients during one day.^[13,18,22]^ However, according to an expert opinion on the interpretation of cutoff values for FC in IBD patients, FC less than 100 μg/g was associated with mucosal remission.^[13]^ In our study, although a positive value of FC was ≥ 50 μg/g, we divided the patients into active and inactive mucosal inflammations based on FC with a 100 μg/g cutoff value. This study showed that the optimal cutoff of the appropriate time interval for dividing the association and non-association between FC and MES was 7 days using an ROC curve with a cutoff level of 200 μg/g for FC. The sensitivity, specificity, accuracy, PPV, and NPV were observed to be the highest at 74.4%, 50.0%, 61.2%, 55.6%, and 70.0%, respectively, compared with the cutoff values of FC 250 or 300 μg/g. Unfortunately, this result did not show statistically significant implications, such as the high values of AUC, sensitivity, and specificity. This may be due to the small sample size and retrospective design of the single-center study with potential bias. Contrary to the study purpose, we could not make a definitive conclusion as the disease activity may be evaluated through an endoscope within 7 days in UC patients with FC scores of 200 μg/g or higher.

Many studies have reported that FC correlates better with endoscopic disease activity than serum inflammatory biomarkers.^[23–26]^ A study reported that UC patients with shorter periods (≤14 days) between the FC measurement and endoscopy, the higher correlation with endoscopic disease activity.^[27]^ On the other hand, there was no significant correlation difference in our patients with shorter intervals (≤7 days) between the FC test and endoscopic evaluation as shown Figure 1. Patients with longer intervals (> 7 days) showed a gradual increase in the FC level correlated with increasing MES, which was not as well captured a that in patients with shorter intervals (≤7 days). In UC patients with shorter intervals, one patient with MES 0 compared with 27 patients with MES1 showed higher FC. This may be explained by the mucosal evaluation in part of the large bowel using sigmoidoscopy, not colonoscopy was performed in only one patient with an MES of 0. Although the AUC according to the time interval (≤7 days) between the FC measurement and endoscopy was not statistically significant, more significant therapeutic plan adjustments were observed in patients with shorter intervals (≤7 days) than in longer intervals (>7 days) in patients with UC. Therefore, these results imply that endoscopy within a shorter interval can help in therapeutic decisions in patients with UC with high FC.

The limitations of our study include its retrospective, single institution with a relatively small sample size. There is much bias against changing therapeutic plans, which made it difficult to evaluate the effect of all covariates influencing therapeutic plan adjustment, due to the limitations of the retrospective analysis. To compensate for these limitations, we are planning a well-designed prospective study to confirm the study purpose and conduct multivariable logistic regression analysis to evaluate the effect of covariates for therapeutic plan adjustment. Endoscopic activity was assessed using only MES because of the major limitation of the retrospective design. The endoscopic index of UC has been reported to be a reliable endoscopic activity index in patients with UC.^[28,29]^ Although MES is not validated, it is the most widely used index.^[17]^ To evaluate endoscopic activity, and the final endoscopic score was determined after two experts independently assessed the endoscopic images and reached an agreement.

In conclusion, endoscopy within 7 days after detecting high calprotectin levels did not adequately reflect the activity of endoscopic disease severity, whereas endoscopy within a shorter interval (≤7 days) in UC patients with high FC can be useful for determining the therapeutic plan.

## Author contributions

**Conceptualization:** Seong Ran Jeon.

**Data curation:** Ho Min Yong, Sung-Jo Park, Tae Hee Lee, Junseok Park, Jin-Oh Kim, Joon Seong Lee, Bong Min Ko, Hyeon Jeong Goong.

**Formal analysis:** Ho Min Yong, Sung-Jo Park, Seong Ran Jeon, Heesu Park, Hyun Gun Kim, Suyeon Park.

**Investigation:** Heesu Park, Junseok Park, Bong Min Ko, Hyeon Jeong Goong.

**Methodology:** Suyeon Park.

**Supervision:** Seong Ran Jeon, Hyun Gun Kim, Jin-Oh Kim, Joon Seong Lee.

**Validation:** Tae Hee Lee, Suyeon Park.

**Writing – original draft:** Ho Min Yong, Sung-Jo Park.

**Writing – review & editing:** Ho Min Yong, Seong Ran Jeon.
